# Observed Workplace Incivility toward Women, Perceptions of Interpersonal Injustice, and Observer Occupational Well-Being: Differential Effects for Gender of the Observer

**DOI:** 10.3389/fpsyg.2016.00482

**Published:** 2016-05-17

**Authors:** Kathi N. Miner, Lilia M. Cortina

**Affiliations:** ^1^Department of Psychology and Women's and Gender Studies Program, Texas A&M UniversityCollege Station, TX, USA; ^2^Departments of Psychology and Women's Studies, University of MichiganAnn Arbor, MI, USA

**Keywords:** workplace incivilty, gender differences and similarities, organizational justice, job satisfaction, turnover intention, organizational trust, safety, fairness

## Abstract

The present study examined perceptions of interpersonal injustice as a mediator of the relationship between observed incivility toward women at work and employees' occupational well-being. We also examined gender of the observer as a moderator of these mediational relationships. Using online survey data from 1702 (51% women; 92% White) employees, results showed that perceptions of injustice partially mediated the relationship between observed incivility toward women and job satisfaction, turnover intentions, and organizational trust. Men reported greater perceptions of injustice than did women the more they observed the uncivil treatment of women at work, and the indirect effects of observed incivility toward women on well-being were stronger for men compared to women. Observed incivility toward women also had direct relationships with the occupational well-being outcomes over and above the impact mediated through injustice, particularly for women. Specifically, observing incivility toward female coworkers directly related to lowered job satisfaction and perceptions of safety for female bystanders. In addition, although both male and female bystanders reported heightened turnover intentions and lowered trust in the organization with higher levels of observed incivility toward women, these relationships were stronger for female than male observers. Our findings both replicate and extend past research on vicarious workplace incivility toward women.

## Introduction

Most research on mistreatment in the workplace has focused on the direct, active, physical types of hostile behavior that occur in work settings (Neuman and Baron, 1997; Barling et al., 2009). More recently, researchers have become interested in lesser, more subtle forms of maltreatment such as rude, disrespectful behavior (Deitch et al., 2003; Dipboye and Halverson, 2004; Cortina, 2008; Jones et al., 2013). One type of behavior in this more recent stream of research is workplace incivility. *Workplace incivility* is defined as workplace behavior that violates norms for mutual respect, is characteristically rude and discourteous, and conveys absence of regard for others (Andersson and Pearson, 1999).

Examples of workplace incivility include interrupting colleagues, addressing others in an inappropriate way, and making jokes at another's expense. Numerous research studies have documented that personal experiences of workplace incivility can interfere with the occupational well-being of targets (Cortina et al., 2001, 2002; Pearson and Porath, 2005; Lim et al., 2008; Cortina and Magley, 2009; Miner and Eischeid, 2012; Miner et al., 2012; Porath and Pearson, 2012).

Although researchers have established a clear link between personal experiences of workplace incivility and detrimental outcomes, less is known about how observers are affected. Andersson and Pearson (1999) theorized that the negative impact of workplace incivility can be felt not only by targets, but also observers. Barling (1996) also argued for the importance of examining the consequences for “secondary victims,” or employees who are vicariously exposed to workplace mistreatment. To date, only a handful of studies have examined the effects of witnessing uncivil workplace behavior (e.g., Porath et al., 2010), including incivility toward certain employees (e.g., women; Miner-Rubino and Cortina, 2004, 2007). This preliminary research shows that observing workplace incivility, including workplace incivility toward women, relates to negative outcomes for observers. Still, more research is needed assessing the consequences of bystander experiences of incivility, particularly incivility targeted at women. Research shows that women are especially likely to experience uncivil treatment at work (Cortina et al., 2001, 2002, 2013; Settles and O'Connor, 2014), making the observed mistreatment of women a potentially common occurrence during the average workday. Research is also needed examining the mechanisms through which uncivil observations affect employee outcomes. Andersson and Pearson (1999) provide one possibility: they theorized perceptions of injustice as a key mediator linking personal experiences of workplace incivility to negative outcomes for employees. We propose that perceived injustice may also mediate the relationship between observing incivility toward women at work and detriments in observers' occupational well-being.

**Figure 1:**
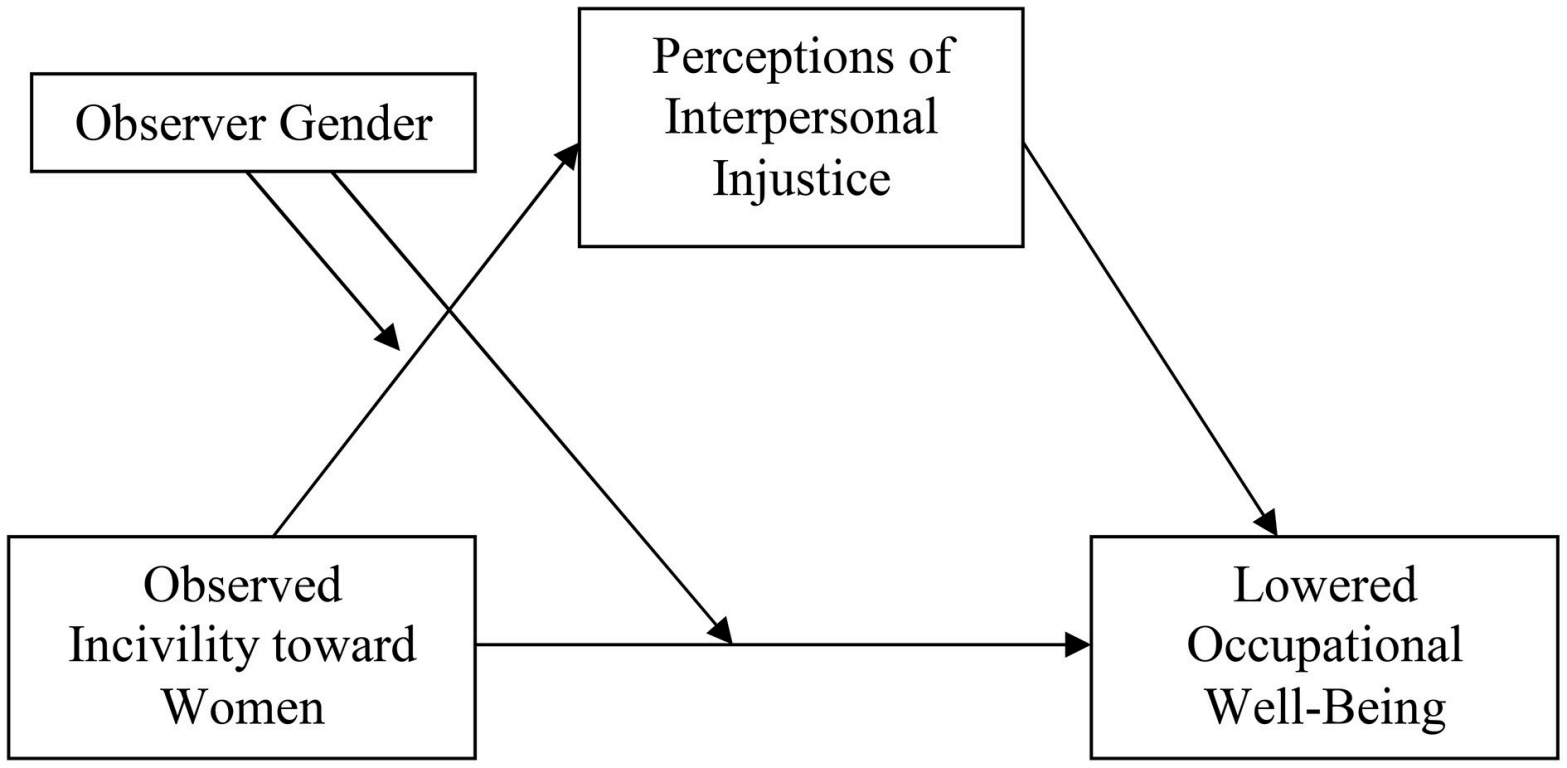
**Proposed moderated mediation model**.

The purpose of the present study is to examine perceptions of injustice as a mediator of the relationship between observed incivility toward women at work and employees' occupational well-being. We predict that perceptions of injustice mediate the relationship between observed workplace incivility toward women and four work-related well-being outcomes: job satisfaction, turnover intentions, organizational trust, and perceptions of safety. We also examine the extent to which these relationships differ for male and female observers and propose that female observers have greater perceptions of injustice and show greater detriments in well-being with higher levels of observed incivility toward female coworkers. We describe the rationale for our hypotheses more fully below. Our proposed moderated mediational model is displayed in Figure 1.

### Observed workplace incivility

Researchers have only begun to examine the consequences of observing incivility toward others in work contexts. Holm (2014) reported that observed workplace incivility related to declines in psychological well-being and job satisfaction and heightened levels of stress for bystanders. Porath et al. (2010) found that witnessing an incident of incivility between employees made customers angry and led to customers ruminating about the incident. It also caused customers to make negative generalizations about employees of the company, the company in general, and future encounters with the company. Reich and Hershcovis (2015) found that observations of workplace incivility led observers to experience heightened negative affect and, in turn, punish the instigator.

Research has also documented negative effects of observing the maltreatment of women at work. For example, Glomb et al. (1997) found that indirect exposure to the sexual harassment of women related to declines in well-being and increases in organizational withdrawal for bystanders. Similarly, Miner-Rubino and Cortina (2004) found that employees who observed incivility toward women at work reported decreased health satisfaction. Miner-Rubino and Cortina (2007) further found that vicarious mistreatment toward women (i.e., observed sexual harassment and incivility) related to declines in psychological well-being, physical health, and job satisfaction, and increases in various organizational withdrawal behaviors (e.g., turnover intentions, organizational commitment) for employees. Other findings have demonstrated that observing misogyny relates to emotional distress for observers (Cunningham et al., 2012). Thus, evidence suggests that all employees in an organization can be harmed by working in a context that tolerates the mistreatment of others, and women specifically, even those who are not direct targets.

While past research has identified various negative outcomes associated with vicarious experiences of incivility and other forms of mistreatment toward women, the mechanisms underlying these relationships remain unclear. Most researchers (e.g., Glomb et al., 1997; Miner-Rubino and Cortina, 2004, 2007) have conceptualized bystander experiences of workplace mistreatment as one type of work stress. The traditional work stress framework is one in which workplace events are “stressors,” individuals' perceptions and interpretations of these events reflect “stress,” and this stress is responsible for declines in well-being (Lazarus and Folkman, 1984). Models of work stress are a useful first step for understanding how working in a negative environment for women affects outcomes, but we sought to identify a more specific mechanism that mediates the relationship between observing incivility toward women at work and occupational well-being outcomes for observers. That is, our research asks *why* observations of women being treated uncivilly can be stressful to employees. We propose that perceptions of interpersonal injustice represent a key pathway through which bystander experiences of incivility toward women influence employee outcomes. That is, we posit that injustice perceptions mediate the relationship between observed incivility toward women at work and declines in observers' occupational well-being.

### Perceptions of interpersonal injustice

Research shows that issues of justice and fairness are important to people in general, in everyday interactions, and in the workplace (Bies, 2001). Employees look to the treatment of their coworkers, both with whom they are directly and indirectly connected, for cues and information about organizational norms regarding fairness and justice (van de Bos and Lind, 2001; Lamertz, 2002). Employees appear to be particularly sensitive to unequal treatment among employees (van de Bos and Lind, 2001). Thus, perceptions of the treatment of coworkers serve as salient signals by which employees determine the degree to which employees are valued by the organization (Tyler and Lind, 1992). According to Lind (2001), a sense of justice and fairness involves feelings of positive regard, respect, social inclusion, and dignity. In contrast, a sense of injustice involves feelings of disrespect, inconsideration, abuse, rudeness, and humiliation (Bies and Moag, 1986; Tyler and Lind, 1992; Bies, 2001).

Most research examining perceptions of justice in organizational settings has focused on distributive and procedural justice. *Distributive justice* refers to perceptions regarding decision outcomes (Adams, 1965; Leventhal, 1976) and *procedural justice* refers to perceptions regarding the processes involved in outcome decisions (Thibaut and Walker, 1975; Leventhal, 1980). Researchers have also begun to examine *interactional justice*, which refers to perceptions regarding interpersonal treatment (Bies and Moag, 1986). Bies and Moag (1986) identified four components of interactional justice: justification (e.g., explaining how decisions were made), truthfulness (e.g., supervisors refraining from deception), respect (e.g., being courteous), and propriety (e.g., refraining from improper and prejudicial remarks). These four components are often collapsed into two separate dimensions: explanations (termed *informational justice*) and sensitivity (termed *interpersonal justice*) (Greenberg, 1993), which have been shown to have independent effects (Greenberg, 1994; Colquitt, 2001; Colquitt et al., 2001). In the present research, we focus specifically on perceptions of interactional injustice (and interpersonal justice in particular), because these justice perceptions are most relevant to how employees might interpret rude, discourteous behavior directed toward female coworkers.

According to Fairness Theory (Folger and Cropanzano, 2001), people judge whether an injustice has occurred through a decision-making process. In this process, individuals cognitively assess how just and fair an incident or action is by looking for signs of regard, respect, social inclusion, and dignity (Tyler and Lind, 1992; Folger and Cropanzano, 2001). Individuals conclude that the incident or action is unjust when they perceive it as disrespectful, inconsiderate, rude, or humiliating (Bies and Moag, 1986; Tyler and Lind, 1992; Bies, 2001). The conclusion that injustice has occurred is theorized to then have important negative consequences for the perceiver. For example, Fairness Theory (Folger and Cropanzano, 2001) predicts that perceptions of injustice will have implications for well-being, and Bies (2001) argued that perceived unjust treatment may directly lead to an employee's discontentment with the organization. Empirical research documents these negative effects. A meta-analysis conducted by Colquitt et al. (2001) reported relationships between interactional injustice perceptions (as well as the other forms of justice) and a host of negative outcomes including decreased job satisfaction and organizational citizenship behaviors. Simons and Roberson (2003) found that aggregate department-level perceptions of interpersonal injustice related to lower levels of supervisor satisfaction and affective organizational commitment, and higher turnover intentions among hotel employees.

Particularly germane to the present study, researchers have also linked perceptions of injustice with workplace mistreatment. Research shows that perceptions of injustice mediate the relationship between sexual harassment and organizational commitment, withdrawal intentions, and job performance (Barling et al., 2001); abusive supervision and subsequent employee aggression (Burton and Hoobler, 2011; Wang et al., 2012); and working in a climate of workplace incivility and intention to remain with the organization (Griffin, 2010). To date, then, research and theory suggest that experiences of mistreatment are related to perceptions of injustice and, as a result, to declines in employees' well-being.

We propose that perceptions of injustice might also mediate the relationship between observed workplace incivility toward women and work-related outcomes for observers. When employees observe the uncivil treatment of women in their workplace, they may conclude that the organization treats some employees unfairly, and may perceive the organization as unjust. By definition, workplace incivility violates standards of respect and dignity and, when observed, could arouse a sense of injustice in bystanders. As a result, bystander employees' occupational well-being may suffer. We examine two forms of occupational well-being assessed in previous studies of observed incivility: job satisfaction and turnover intentions. We also examine two additional forms of well-being not yet assessed but that may be related to observing women being treated uncivilly at work: organizational trust and perceptions of safety. Indeed, organizational trust has been linked with workplace incivility (Gill and Sypher, 2009; Miner-Rubino and Reed, 2010) and perceptions of justice (Colquitt et al., 2001; Kale, 2013; Tlaiss and Elamin, 2015) in the literature. Research also suggests that experiences of incivility (Miner and Eischeid, 2012; Porath and Pearson, 2012) and perceptions of justice (Taxman and Gordon, 2009) predict perceptions of safety in organizations. This research and theory led us to the following hypotheses:

*Hypothesis 1*: Observed incivility toward women is positively related to perceptions of injustice.
*Hypothesis 2*: Perceptions of injustice are negatively related to observers' occupational well-being (lowered job satisfaction, organizational trust, and perceptions of safety, and higher turnover intentions).
*Hypothesis 3*: Perceptions of injustice mediate the relationship between observed incivility toward women and observers' occupational well-being.

### Observer gender as a moderator

We propose above that employees will be negatively affected by observing incivility toward women at work and that perceptions of injustice mediate this relationship. We further propose that these relationships will be stronger for women compared to men. We base this proposition in part on similarity/attraction theory (Byrne, 1971) which proposes that people are attracted to and feel a common fate with those who are similar to them. Thus, women should be more affected than men when they observe incivility toward other women simply because they feel more connected to their female coworkers. Preliminary research supports this idea (Miner and Eischeid, 2012). Research findings have also documented that women may be more sensitive to and offended by interpersonal mistreatment in work contexts compared to men (Rotundo et al., 2001; Young et al., 2003; Montgomery et al., 2004; Escartín et al., 2011). Based on this research and theory, we formed the following hypotheses:

*Hypothesis 4*: The positive relationship between observed incivility toward women and perceptions of injustice is moderated by observer gender such that the relationship is stronger for women compared to men.
*Hypothesis 5*: The negative relationship between observed incivility toward women and observers' occupational well-being is moderated by observer gender such that the relationship is stronger for women compared to men.
*Hypothesis 6*: The indirect effect of observed incivility toward women on observers' occupational well-being through perceptions of injustice is stronger for women compared to men.

## Method

### Participants and procedure

All employees (i.e., faculty and staff) at a small northwestern public university (*N* = 2773) were invited to participate in a “respectful climate survey” which had been approved by the researchers' and participants' Institutional Review Boards. Employees who had access to a computer were asked to complete the survey on-line; those without access were mailed a paper survey. To maximize return rates, employees received advance notices, invitations, and reminders about the survey from the university president (Dillman et al., 2008). On the first page of the survey, instructions described the purpose of the study, assured confidentiality, and reminded employees that they could skip any items. Respondents had the opportunity to win gift certificates as a further participation incentive. Using these procedures, 1843 participants returned the questionnaire (67% response rate). Because of extensive missing data, 141 surveys were excluded. Thus, the total number of participants with usable data was 1702 (51% women). Of those, 1390 completed the survey on-line (82%); the remaining participants mailed in their completed paper survey.

Participants ranged in age from 20 to 75 (*M* = 43.63, *SD* = 10.24), and most were White (92%), employed full-time (93%), and had at least some college or a college degree (94%). They had worked for the university for an average of 10 years. Their job classifications were as follows: 31% were employed in technical/paraprofessional/skilled craft positions, 20% full/associate professor, 16% non-faculty exempt, 12% assistant professor/lecturer/instructor, 12% secretarial/clerical, 3% administrator, 3% irregular help, and 2% service/maintenance.

### Measures

The survey included a number of multi-item scales; most relevant to the current study were measures of observed incivility toward women in the work environment, perceptions of interpersonal injustice, and occupational well-being (i.e., job satisfaction, turnover intentions, organizational trust, and perceptions of safety). Construction of the larger survey focused on minimizing response bias and utilizing valid and reliable measures. For example, well-being measures appeared before questions assessing uncivil observations to allow for an unbiased assessment of employee functioning. All items were scored such that higher values reflect higher levels of the underlying construct.

#### Observed incivility toward women

We assessed observed incivility toward women with six items based on the Workplace Incivility Scale (WIS; Cortina et al., 2001). Participants rated the items on a response scale from 0 (*never)* to 2 (*more than once or twice*), asking how often in the past year they had observed disrespectful, rude, and condescending behavior directed toward female employees. Behaviors included “speak in a condescending or patronizing manner,” “treat in a disrespectful or discourteous manner,” and “ignore, fail to listen to, or interrupt.” This scale showed adequate reliability (Cronbach's α = 0.81).

#### Perceptions of interpersonal injustice

Perceptions of interpersonal injustice were measured with the Perceptions of Fair Interpersonal Treatment Scale (PFIT; Donovan et al., 1998). This scale assessed employees' perceptions of the interpersonal norms in their workplace; thus, this scale specifically measures interpersonal justice (Greenberg, 1993). Participants indicated whether eight statements characterize their workplace, using a “no,” “?,” “yes” response format. Example items for this measure include, “Employees are treated fairly,” (reverse-coded) and “Employees put each other down.” Together the items formed a reliable scale (α = 0.88). This scale was coded so that higher scores represented greater perceptions of interpersonal injustice.

#### Occupational well-being

*Job satisfaction* was measured with items from the Michigan Organizational Assessment Questionnaire (Cammann et al., 1979). Respondents indicated on a scale from 1 (*strongly disagree*) to 7 (*strongly agree*) the extent to which each of three statements characterized their work: “All in all, I am satisfied with my job,” “In general, I like working here,” and “In general, I don't like my job” (reverse-coded). This measure showed acceptable reliability (α = 0.83).

*Turnover intentions* were measured with Porter et al.'s (1976) 2-item measure. Participants were asked to indicate, on a scale from 1 (*strongly disagree*) to 7 (*strongly agree*), their level of agreement with the statements, “I often think about quitting this job” and “I will probably look for a new job during the next year.” Coefficient alpha for this measure was 0.76.

*Organizational trust* was measured with five items from the Interpersonal Trust at Work Scale (Cook and Wall, 1980). This instrument assesses the extent to which participants ascribe good intentions to and have confidence in the words and actions of organizational leaders. Example items include “I feel quite confident that the institution will always try to treat me fairly,” and “The administration at this institution is sincere in its attempt to meet the employees' point of view.” Items were rated on a seven-point scale ranging from 1 (*strongly disagree*) to 7 (*strongly agree*). The items in this scale showed good reliability (α = 0.88).

*Perceptions of safety* were measured with three items assessing participants' perceptions of being safe on campus. Items included “I feel safe from physical attack at this university,” “When walking to transportation (for example, my car, the bus) at night, I worry about my safety,” (reverse-coded) and “I feel safe from physical attack when working at night.” Items were rated on a 7-point scale ranging from 1 (*strongly disagree*) to 7 (*strongly agree*). Coefficient alpha for this measure was also adequate (α = 0.76).

#### Control variable

Previous research has demonstrated that *dispositional negative affectivity* may bias individuals' responses to items in a survey, such that they answer items with a pessimistic slant (Levin and Stokes, 1989; Judge and Hulin, 1993). Research also shows that individuals with a negative dispositional stance are more likely to have an unconscious attentional bias for negative stimuli, such as the mistreatment of others (Segerstrom, 2001). Because of these reasons, participants also completed a measure of dispositional negative affectivity to serve as a control in the analyses. This dispositional stance was measured with the Life Orientation Test (Scheier and Carver, 1985), which assesses dispositional optimism, or the tendency to expect favorable outcomes. We scored this scale such that higher scores represent lower optimism, or higher negativity. Instructions asked respondents the degree to which they agree or disagree with eight statements, using a scale from 1 (*strongly disagree*) to 7 (*strongly agree*). Example items include, “If something can go wrong for me it will” and “Every cloud has a silver lining” (reverse-scored). This measure showed good internal reliability (α = 0.87).

## Results

Table 1 presents the means, standard deviations, and intercorrelations for all variables in the present study. Observed incivility toward women was correlated with perceptions of interpersonal injustice and all four occupational well-being variables. In addition, perceptions of injustice correlated with the occupational well-being variables and the occupational well-being variables were all intercorrelated. Finally, negative affectivity was associated with perceptions of injustice and the occupational well-being variables, corroborating our decision to include it as a covariate in the analyses.

**Table 1:** **Means, standard deviations, and intercorrelations among all study variables**.

**Variable**	**M**	**SD**	**1**	**2**	**3**	**4**	**5**	**6**
1. Observed incivility toward women	0.39	0.41						
2. Perceptions of interpersonal injustice	1.47	0.61	0.40^***^					
3. Job satisfaction	5.78	1.13	−0.28^***^	−0.52^***^				
4. Turnover intentions	2.95	1.70	0.27^***^	0.48^***^	−0.67^***^			
5. Organizational trust	4.54	1.29	−0.34^***^	−0.56^***^	0.53^**^	−0.50^***^		
6. Perceptions of safety	5.45	1.20	−0.14^***^	−0.10^***^	0.10^**^	0.08^**^	0.12^***^	
7. Negative affectivity	2.68	0.90	0.04	0.21^***^	−0.31^**^	0.25^***^	−0.28^***^	−0.11^***^

** *p < 0.01*,

*** *p < 0.001*.

We tested the hypotheses using the procedures outlined by Hayes (2013) for examining conditional indirect effects in moderated mediational models; the simple mediation results appear in Table 2 and the moderated mediation results appear in Tables 3–6. Consistent with Hypothesis 1, observed incivility toward women was positively related to perceptions of interpersonal injustice. Providing support for Hypothesis 2, injustice perceptions were negatively related to occupational well-being (lower job satisfaction, organizational trust, and perceptions of safety, and higher turnover intentions) for observers. Partially supporting Hypothesis 3, the negative effect of observed incivility toward women on observers' job satisfaction, turnover intentions, and organizational trust was reduced, though still significant, when perceptions of injustice was included in the model. Perceptions of injustice did not mediate the relationship between observing incivility toward women at work and perceptions of safety (see Table 2).

**Table 2:** **Simple mediation results**.

	**Mediator**		**Occupational well-being variables**							
**Predictor**	**Perceptions of Injustice**		**Job Satisfaction**		**Turnover Intentions**		**Organizational Trust**		**Perceptions of Safety**	
	***B***	***SE***	***B***	***SE***	***B***	***SE***	***B***	***SE***	***B***	***SE***
Negative affectivity (control)	0.13^***^	0.02	−0.27^***^	0.03	0.30^***^	0.04	−0.25^***^	0.03	−0.13^**^	0.04
Observed incivility toward women	0.61^***^	0.03	−0.25^***^	0.07	0.46^***^	0.10	−0.46 ^***^	0.07	−0.36^***^	0.08
Perceptions of Injustice	–		−0.84^***^	0.04	1.14^***^	0.07	−0.98^***^	0.05	−0.05	0.06
*R^2^*	0.20^***^		0.33^***^		0.27^***^		0.35^***^		0.03^***^	
Bootstrap Indirect Effects (through Perceptions of Injustice)			***B (SE)*** **95% CI**		***B (SE)*** **95% CI**		***B (SE)*** **95% CI**		***B (SE)*** **95% CI**	
			−0.48^*^(0.05) −0.58, −0.39		0.65^*^(0.06)0.54, 0.78		−0.55^*^(0.05) −0.65, −0.46		−0.03(0.04) −0.11, 0.04	

*CI, confidence interval*.

* *p < 0.05*,

** *p < 0.01*,

*** *p < 0.001*.

**Table 3:** **Moderated mediation analysis for observed incivility toward women, perceptions of interpersonal injustice, observer gender, and job satisfaction**.

**Predictor**	***B***	***SE***	***t***
**MEDIATOR—PERCEPTIONS OF INJUSTICE**			
Control: Negative affectivity	0.13	0.02	8.15^***^
Predictor: Observed incivility toward women	0.61	0.03	17.73^***^
Moderator: Observer gender	0.02	0.02	1.07
Interaction: Observed incivility × Observer gender	0.09	0.03	2.52^*^
*R^2^*	0.21		
**OUTCOME—JOB SATISFACTION**			
Control: Negative affectivity	−0.27	0.03	−10.03^***^
Mediator: Perceptions of injustice	−0.84	0.04	−19.88^***^
Predictor: Observed incivility toward women	−0.22	0.06	−3.52^**^
Moderator: Observer gender	−0.04	0.03	−1.34
Interaction: Observed incivility × Observer gender	0.11	0.06	1.85^†^
*R^2^*	0.33		
**Direct and Indirect Effects**	***B***	***SE***	**95% CI**
**CONDITIONAL DIRECT EFFECTS ON JOB SATISFACTION**			
Men	−0.11	0.10	−0.30, 0.07
Women	−0.33^***^	0.08	−0.48, −0.18
**CONDITIONAL INDIRECT EFFECTS ON**			
**JOB SATISFACTION (THROUGH PERCEPTIONS OF INJUSTICE)**			
Men	−0.59^*^	0.07	−0.73, −0.46
Women	−0.44^*^	0.05	−0.55, −0.34
Index of moderated mediation	−0.15^*^	0.07	−0.21, −0.01

*CI, confidence interval*.

† *p < 0.10*,

* *p < 0.05*,

** *p < 0.01*,

*** *p < 0.001*.

**Table 4:** **Moderated mediation analysis for observed incivility toward women, perceptions of interpersonal injustice, observer gender, and turnover intentions**.

**Predictor**	***B***	***SE***	***t***
**MEDIATOR—PERCEPTIONS OF INJUSTICE**			
Control: Negative affectivity	0.13	0.02	8.15^***^
Predictor: Observed incivility toward women	0.61	0.03	17.73^***^
Moderator: Observer gender	0.02	0.02	1.07
Interaction: Observed incivility × Observer gender	0.09	0.03	2.52^*^
*R^2^*	0.21		
**OUTCOME—TURNOVER INTENTIONS**			
Control: Negative affectivity	0.29	0.04	6.94^***^
Mediator: Perceptions of injustice	1.14	0.07	16.86^***^
Predictor: Observed incivility toward women	0.45	0.10	4.41^***^
Moderator: Observer gender	0.12	0.05	2.62^**^
Interaction: Observed incivility × Observer gender	−0.17	0.09	1.87^†^
*R^2^*	0.27		
**Direct and Indirect Effects**	***B***	***SE***	**95% CI**
**CONDITIONAL DIRECT EFFECTS ON TURNOVER INTENTIONS**			
Men	0.27^†^	0.16	−0.03, 0.57
Women	0.61^***^	0.12	0.38, 0.85
**CONDITIONAL INDIRECT EFFECTS ON TURNOVER INTENTIONS**			
**(THROUGH PERCEPTIONS OF INJUSTICE)**			
Men	0.79^*^	0.09	0.62, 0.98
Women	0.59^*^	0.07	0.46, 0.74
Index of moderated mediation	0.20^*^	0.10	0.01, 0.39

*CI, confidence interval*.

† *p < 0.10*,

* *p < 0.05*,

** *p < 0.01*,

*** *p < 0.001*.

**Table 5:** **Moderated mediation analysis for observed incivility toward women, perceptions of interpersonal injustice, observer gender, and organizational trust**.

**Predictor**	***B***	***SE***	***t***
**MEDIATOR—PERCEPTIONS OF INJUSTICE**			
Control: Negative affectivity	0.13	0.02	8.12^***^
Predictor: Observed incivility toward women	0.61	0.03	17.76^***^
Moderator: Observer gender	0.02	0.02	1.07
Interaction: Observed incivility × Observer gender	0.09	0.03	2.58^*^
*R^2^*	0.20		
**OUTCOME—ORGANIZATIONAL TRUST**			
Control: Negative affectivity	−0.25	0.03	−8.38^***^
Mediator: Perceptions of injustice	−0.97	0.05	−20.42^***^
Predictor: Observed incivility toward women	−0.43	0.07	−6.07^***^
Moderator: Observer gender	−0.07	0.03	−2.15^*^
Interaction: Observed incivility × Observer gender	0.16	0.07	2.47^*^
*R^2^*	0.36		
**Direct and indirect effects**	***B***	***SE***	**95% CI**
**CONDITIONAL DIRECT EFFECTS ON ORGANIZATIONAL TRUST**			
Men	−0.27^*^	0.11	−0.48, −0.06
Women	−0.58^***^	0.08	−0.76, −0.43
**CONDITIONAL INDIRECT EFFECTS ON ORGANIZATIONAL TRUST**			
**(THROUGH PERCEPTIONS OF INJUSTICE)**			
Men	−0.68^*^	0.08	−0.85, −0.53
Women	−0.51^*^	0.06	−0.62, −0.40
Index of moderated mediation	−0.18^*^	0.08	−0.33, −0.01

*CI, confidence interval*.

* *p < 0.05*,

*** *p < 0.001*.

**Table 6:** **Moderated mediation analysis for observed incivility toward women, perceptions of interpersonal injustice, observer gender, and perceptions of safety**.

**Predictor**	***B***	***SE***	***t***
**MEDIATOR—PERCEPTIONS OF INJUSTICE**			
Control: Negative affectivity	0.13	0.02	8.27^***^
Predictor: Observed incivility toward women	0.61	0.04	17.51^***^
Moderator: Observer gender	0.02	0.02	1.04
Interaction: Observed Incivility × Observer Gender	0.09	0.04	2.50^*^
**OUTCOME—PERCEPTIONS OF SAFETY**			
Control: Negative affectivity	−0.19	0.03	−6.25^***^
Mediator: Perceptions of injustice	−0.14	0.05	−2.68^**^
Predictor: Observed incivility toward women	−0.07	0.07	0.85
Moderator: Observer gender	0.54	0.03	16.58^***^
Interaction: Observed incivility × Observer gender	0.13	0.07	1.94^†^
**Direct and indirect effects**	***B***	***SE***	**95% CI**
**CONDITIONAL DIRECT EFFECTS ON PERCEPTIONS OF SAFETY**			
Men	−0.07	0.11	−0.15, 0.28
Women	−0.19^*^	0.08	−0.36, −0.02
**CONDITIONAL INDIRECT EFFECTS ON PERCEPTIONS OF SAFETY**			
**(THROUGH PERCEPTIONS OF INJUSTICE)**			
Men	−0.10	0.04	−0.14, −0.00
Women	−0.07	0.03	−0.18, −0.00
Index of moderated mediation	−0.02	0.02	−0.07, −0.00

*CI, confidence interval*.

† *p < 0.10*,

* *p < 0.05*,

** *p < 0.01*,

*** *p < 0.001*.

**Figure 2:**
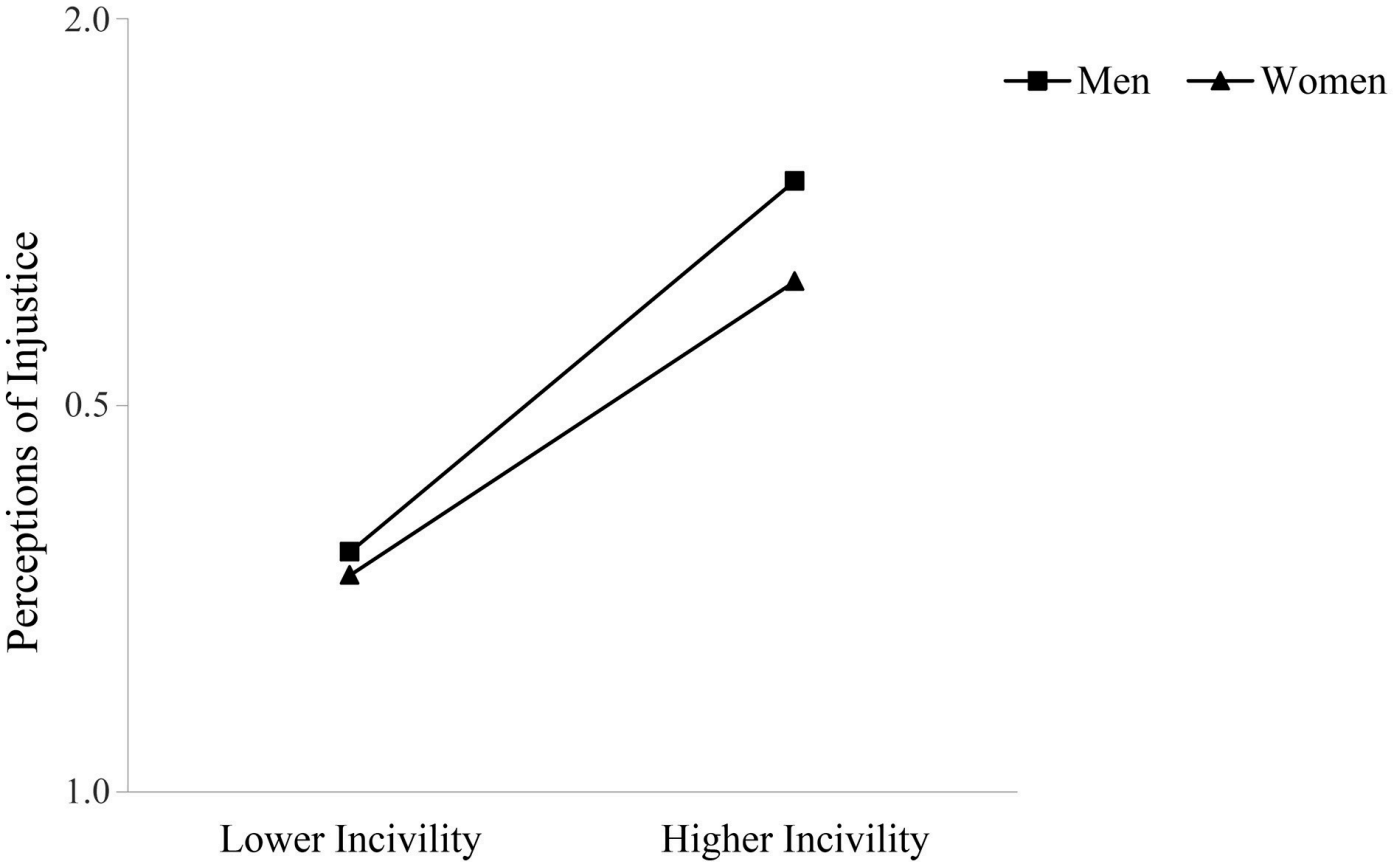
**Interaction of observed incivility toward women and observer gender on perceptions of interpersonal injustice**.

Hypothesis 4, that the positive relationship between observed incivility toward women and perceptions of injustice would be moderated by observer gender such that the relationship would be stronger for women, was not supported. As shown in Figure 2, although both women and men reported greater perceptions of injustice with higher levels of observed incivility toward women at work, this relationship was especially pronounced for male compared to female observers.

**Figure 3:**
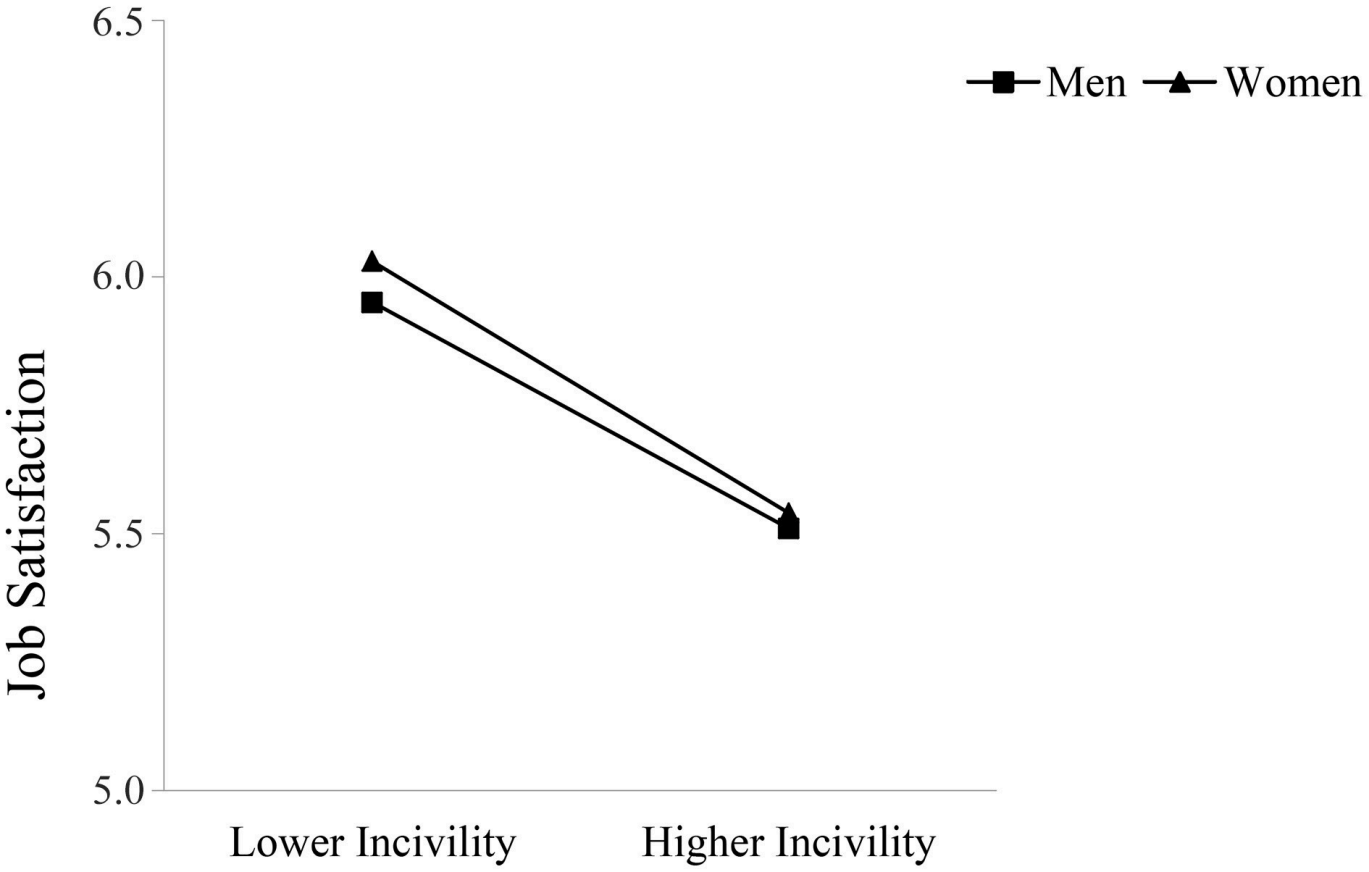
**Interaction of observed incivility toward women and observer gender on job satisfaction**.

**Figure 4:**
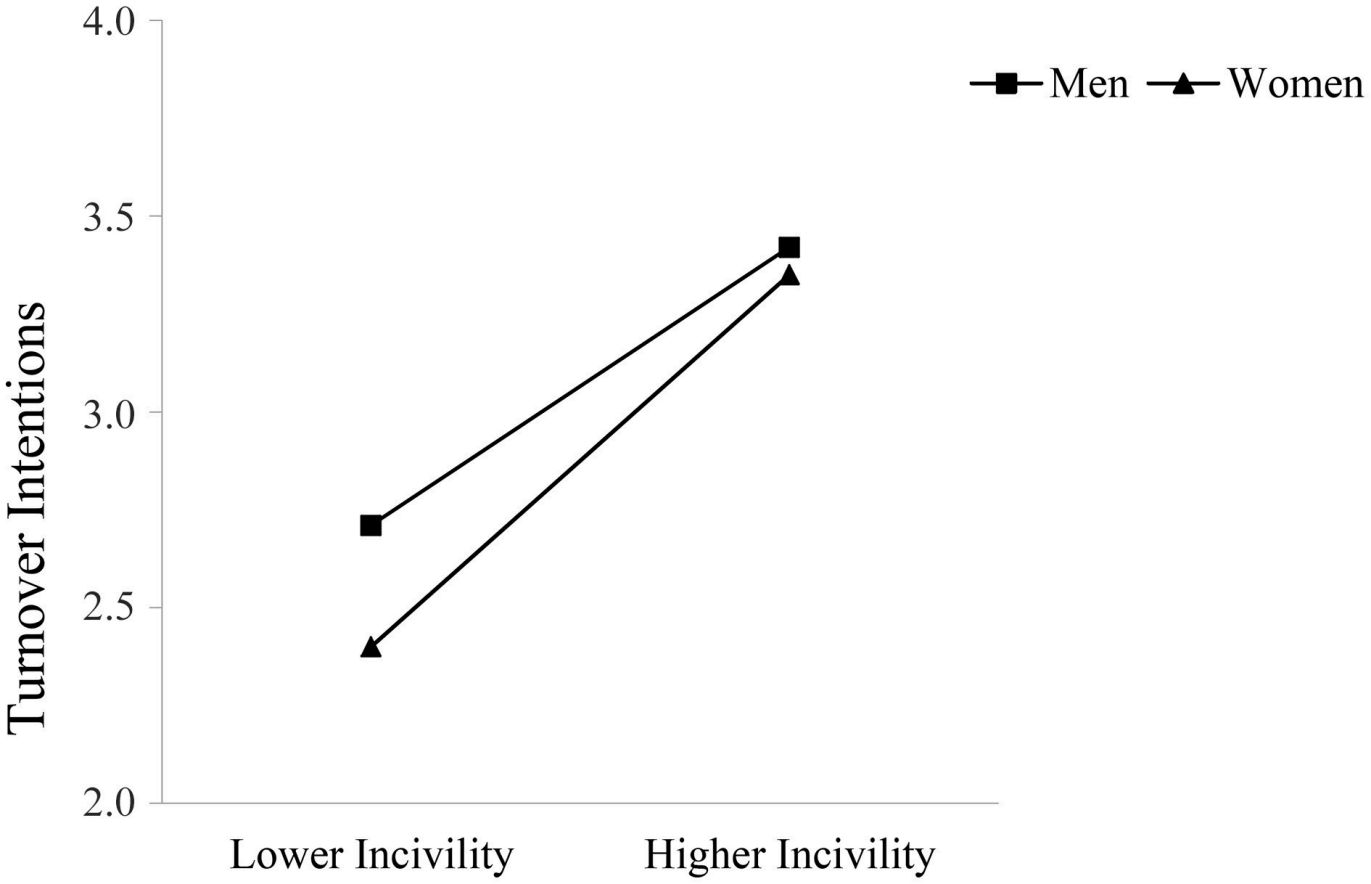
**Interaction of observed incivility toward women and observer gender on turnover intentions**.

**Figure 5:**
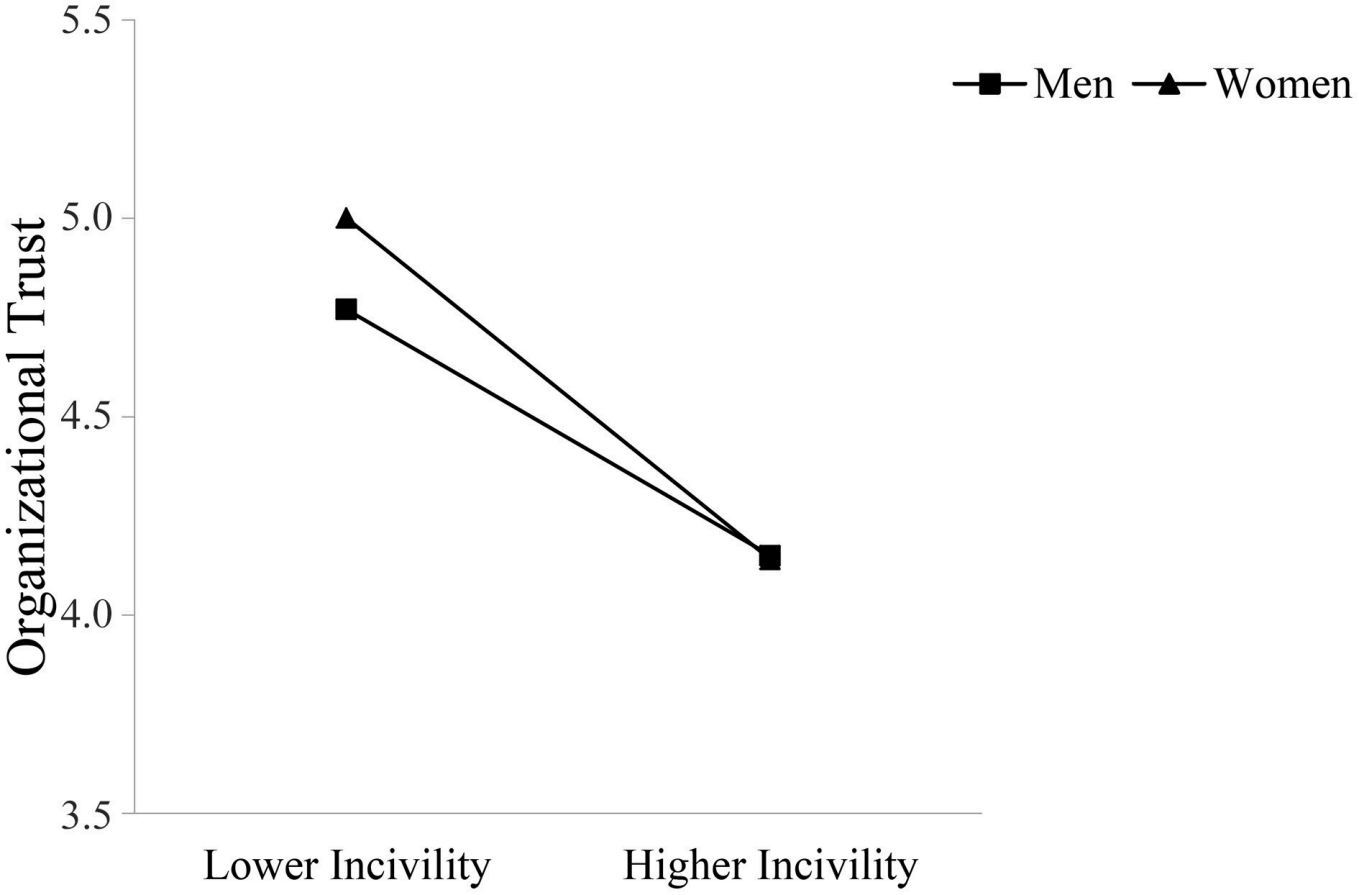
**Interaction of observed incivility toward women and observer gender on organizational trust**.

**Figure 6:**
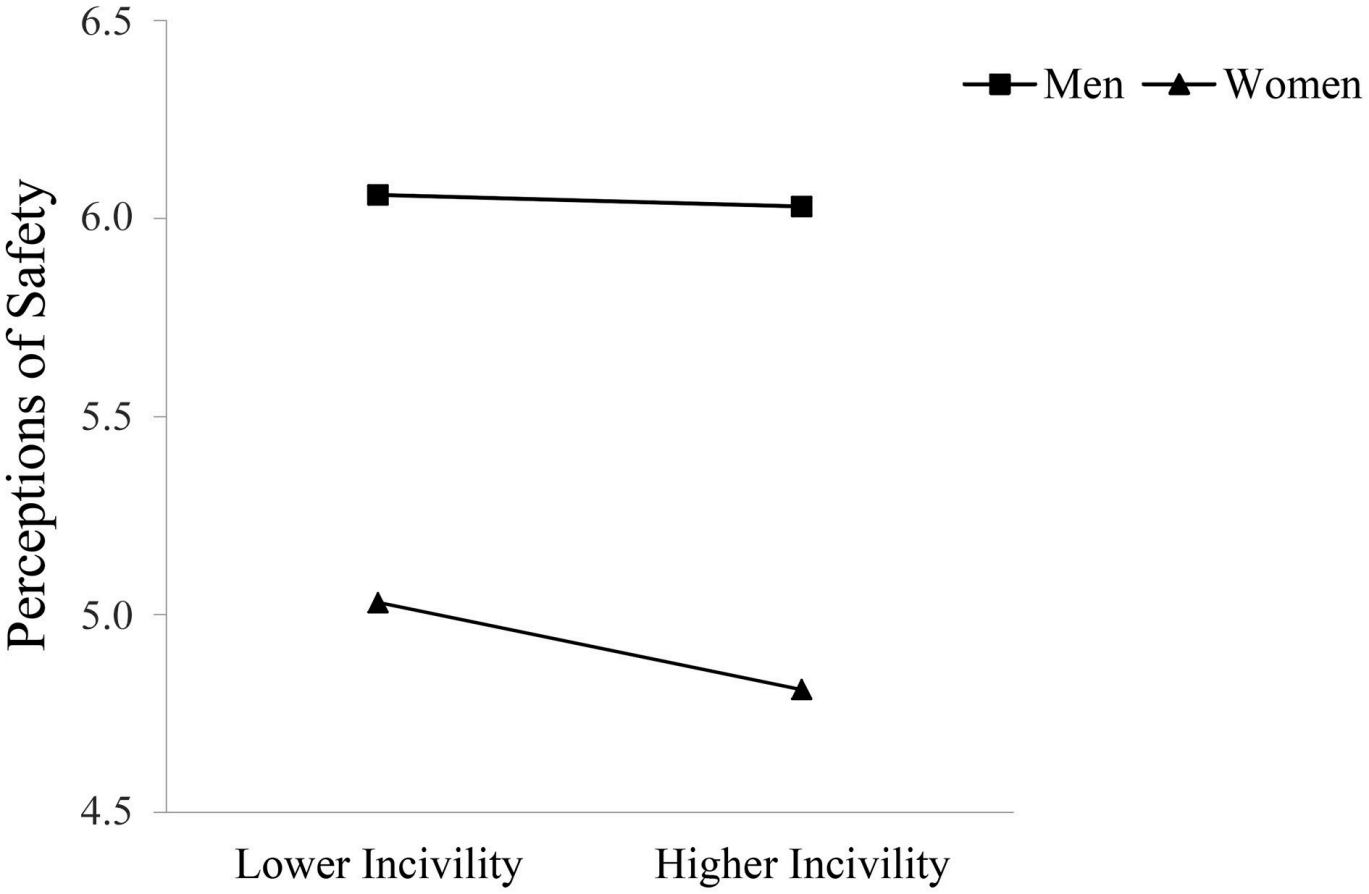
**Interaction of observed incivility toward women and observer gender on perceptions of safety**.

Supporting Hypothesis 5, observer gender moderated the relationship between observed incivility toward women and occupational well-being such that this relationship was stronger for women compared to men. Specifically, women reported lower job satisfaction and perceptions of safety the more they observed incivility toward other women at work; these direct relationships were not significant for men. Men did report greater turnover intentions and lower organizational trust with higher levels of observed incivility toward female coworkers; however, these relationships were again stronger for female observers. Figures 3–6 graphically display the findings for Hypothesis 5. As shown in Figures 3, 6, women reported being less satisfied with their job and feeling less safe with higher levels of observed incivility toward women; these effects were not significant for men. As demonstrated in Figures 4, 5, men reported significantly higher intentions of quitting and lower trust in the university with higher levels of observed incivility toward women; however, these effects were again more pronounced for female observers.

Finally, in Hypothesis 6, we predicted that the indirect effect of observed incivility toward women on observers' occupational well-being, through perceptions of interpersonal injustice, would be stronger for women compared to men. This hypothesis was not supported. Rather, the indirect effect of observed incivility on job satisfaction, turnover intentions, and organizational trust was stronger for men compared to women (recall that perceptions of injustice did not mediate the observed incivility to perceptions of safety relationship). Moreover, as demonstrated by the index of moderated mediation (Hayes, 2015), each moderated mediational model was statistically different from zero providing evidence that the conditional indirect effects for men and women are significantly different from each other, indicating moderated mediation.

## Discussion

The purpose of the present study was to examine perceptions of interpersonal injustice as a mediator of the relationship between observed incivility toward women at work and bystander employees' job satisfaction, turnover intentions, organizational trust, and perceptions of safety. We also examined the extent to which observer gender moderated these mediational relationships. Results revealed that perceptions of interpersonal injustice partially mediated the relationship between observed incivility toward women and job satisfaction, turnover intentions, and organizational trust. These findings suggest that employee perceptions of justice and fairness may be a key mechanism through which vicarious mistreatment interferes with employee well-being. That is, this research elucidates potential reasons why observing the mistreatment of women in the workplace might be stressful for employees, triggering declines in job satisfaction, increased thoughts about leaving the organization, and lowered trust in the organization.

Interestingly, men (not women, as hypothesized) reported greater perceptions of injustice the more they observed the uncivil treatment of women at work, and the indirect effects of observed incivility toward women on well-being were stronger for men compared to women. Injustice perceptions, then, appear to be an especially important factor undergirding the relationship between witnessing uncivil treatment toward female coworkers and detriments in well-being for men. This finding suggests that observing the workplace mistreatment of demographically dissimilar coworkers plays a critical role in shaping perceptions of interpersonal justice and fairness in the larger organizational context. That is, although employees may use the interpersonal treatment of demographic in-group members to form perceptions of the workplace environment, the interpersonal treatment of demographic out-group members may be a particularly important factor in the construction of those perceptions. Indeed, research shows that employees look to the treatment of their coworkers for cues about organizational norms regarding fairness and justice (van de Bos and Lind, 2001; Lamertz, 2002). Our research extends this past research and suggests that the treatment of out-group members may be particularly informative. These more global negative perceptions may in turn drive detriments in occupational well-being.

Observed incivility toward women also showed direct relationships with the occupational well-being outcomes over and above the impact mediated through injustice, particularly for women. Specifically, observing incivility toward female coworkers directly related to lowered job satisfaction and lessened perceptions of safety for female bystanders. In addition, although both male and female bystanders reported heightened intentions to turnover and lowered trust in the organization with higher levels of observed incivility toward women, these relationships were stronger for female than male observers. These findings replicate past research linking observed hostility toward women and job satisfaction and turnover intentions (Miner-Rubino and Cortina, 2004, 2007), and suggest that two additional aspects of occupational well-being not addressed by previous studies—organizational trust and perceptions of safety—are influenced by bystander experiences of incivility toward women at work. Thus, regardless of whether an employee perceives incivility toward women to be unfair, exposure to repeated uncivil events “wears down” the employee, triggering negative well-being.

Our findings also extend past findings by documenting gender of the observer as an important boundary condition under which vicarious incivility toward women affects others. That such observations predicted declines in organizational trust (for both men and women) and perceptions of safety (for women) suggests that witnessing incivility toward women at work has far-reaching consequences, affecting not only employees' satisfaction with and intention to stay in their job, but also how they view the organization as a whole. These findings buttress those of Porath et al. (2010) who found that witnessing incivility between employees negatively influenced customers' attitudes toward the company. Importantly, the links between observed incivility, injustice, and outcomes remained even when controlling for negative affectivity—operationalized as dispositional pessimism. As such, the relationships between observed incivility, perceptions of injustice, and well-being cannot be attributed to an employee's enduring tendency to perceive the world in a negative light.

### Future research directions

There are numerous possibilities for future research in this area. First, there are likely other mediators that help explain the relationship between vicarious gender-based incivility and observers' well-being outcomes. As such, one possibility for future research is to examine other mediators of this relationship. Perceptions of interpersonal injustice represent the “cold,” cognitive response to observed mistreatment. Witnessing the rude treatment of women at work is also likely to have “hot,” emotional components, such as anger (at the perpetrator), sadness (for the plight of the victim), or fear (of being the next target). Emotional reactions could be additional mechanisms that drive the harms of observed incivility toward women. Indeed, past research has identified a link between observed incivility and emotional reactions (Porath et al., 2010; Cunningham et al., 2012; Miner and Eischeid, 2012; Reich and Hershcovis, 2015). Such emotional reactions may be especially germane to understanding why women's vicarious experiences of incivility toward women relate to negative outcomes given that perceptions of injustice more strongly related to declines in well-being for male compared to female observers. Examining emotional reactions as a mechanism linking observed incivility toward women and declines in observers' occupational well-being represents a fruitful avenue for future inquiry.

Another interesting question for future research surrounds the observer's behavior following observations of the mistreatment of women. The current study speaks to outcomes related to the observer's job (e.g., thoughts of quitting) and the organization (e.g., organizational trust). It would be interesting to understand observer outcomes that are more behavioral. For example, upon witnessing the abuse, does the observer intervene, report the behavior to management, or dismiss the situation as trivial? Moreover, do these reactions depend upon the observer's perceptions of injustice? Bowes-Sperry and O'Leary-Kelly (2005) offered a comprehensive typology of observer intervention strategies, proposing factors that may increase the likelihood of observers intervening in cases of harassment. They argued that observers will feel the most personal responsibility to intervene when the target of the mistreatment shares an important demographic feature with the observer, such as gender. Moreover, observer intervention could be an important resource for organizations trying to create a hospitable environment for women, as uncivil and misogynistic conduct often goes unreported. These are interesting possibilities for future research to address.

It would also be fruitful for future studies to examine the extent to which our findings extend to observing incivility toward other groups in the workplace. That is, do observers report being less satisfied with work, thinking more about quitting, having less trust in the organization, and feeling unsafe when they observe underrepresented minorities, gay men and lesbians, or older workers treated rudely at work? Further, are such observations mediated by perceptions of injustice, or are there other mechanisms that would better explain the relationship between witnessing incivility toward these specific groups and observer well-being? Such relationships may also depend on whether the observer is from the same demographic group as the target of incivility, as we found for some relationships in the present study. At the same time, our finding that men perceived incivility toward women as more unjust than did women suggests that individual differences of observers, such as social dominance orientation, empathy, or tolerance for diversity may be more relevant factors that determine how observers are affected by vicarious incivility toward coworkers than shared demography. Clearly, there are numerous possibilities for advancing research examining how and why bystander experiences of workplace incivility toward women affect employees.

### Study limitations

This research has a number of limitations that should be acknowledged. First, our findings were based on employees working in a very unique organizational context—academia—and concerns may arise about the generalizability of results. Indeed, university settings have employees in both white (e.g., administrators) and blue collar (e.g., janitors) job positions with varying levels of autonomy, flexibility, and status. Universities also tend to have hierarchical gender and race structures with men and whites occupying the most prestigious, powerful positions within in the organization. Therefore, our findings may not easily generalize to organizations with less occupational diversity or that are numerically dominated by one gender or race (e.g., elementary schools, hair salons). Our findings are most applicable, then, to employment contexts with characteristics similar to our sample.

A second limitation is the cross-sectional nature of these data, which renders causal inferences tentative. As such, we cannot say definitively that observing the mistreatment of women at work causes perceptions of injustice which, in turn, cause occupational impairment. However, research on longitudinal models of harassment suggests that these outcomes follow from personal experiences of mistreatment (Glomb et al., 1999; Munson et al., 2000); it seems plausible that they might also follow from vicarious exposure to gender-based mistreatment. In addition, following the recent arguments of Fiedler et al. (2011) and Tate (2015), true tests of mediation require a conceptually time-ordered relationship between predictor, mediator, and outcome that assumes a causal path in order for the technique to work. Given these advancements in thinking regarding testing mediation, our results should be interpreted with caution until they can be replicated longitudinally or experimentally. Indeed, it is equally viable that observed incivility, perceptions of interpersonal injustice, and observer gender combine to produce a joint (moderated) effect on lowered occupational well-being rather than the moderated mediational model assessed in the present study. Nevertheless, we examined our proposed model to better situate our research within the current theoretical and empirical literature on workplace incivility. We encourage future research to examine our proposed model over time such that observed incivility, perceptions of injustice, and occupational well-being are assessed in progression during a specific incident.

## Conclusion

Although misogyny can be subtle, its effects are quite clear. Women targeted with pervasive disrespect ultimately become dissatisfied with their job and exit at higher rates. These negative effects, according to the current study, extend to bystanders as well. We also elucidated the role of injustice in this process, helping explain how and why vicarious gender-based mistreatment can be harmful. Although seemingly trivial, these everyday incivilities can make for an unjust work environment, having a negative impact on not only victims, but also bystanders and likely whole organizations as well.

## Author contributions

Both KM and LC worked on the writing of the manuscript and helped collect the data. KM conducted the statistical analyses.

### Conflict of interest statement

The authors declare that the research was conducted in the absence of any commercial or financial relationships that could be construed as a potential conflict of interest.
